# Associations Between Quality of Life, Functional Fitness, Body Composition, and Accelerometer-Measured Physical Activity in Postmenopausal Women: A Cross-Sectional Study

**DOI:** 10.3390/sports14020054

**Published:** 2026-02-03

**Authors:** André Schneider, Flavio Teresinho Mendonça, Letícia Amaral, Tiago M. Barbosa, Pedro Forte, José E. Teixeira, António M. Monteiro

**Affiliations:** 1 Department of Sports Sciences, Instituto Politécnico de Bragança, 5300-253 Bragança, Portugal; andrecschneider@gmail.com (A.S.); flavio.t.mendonca@gmail.com (F.T.M.); barbosa@ipb.pt (T.M.B.); pedromiguelforte@gmail.com (P.F.); 2 Research Centre for Active Living and Wellbeing (LiveWell), Instituto Politécnico de Bragança, 5300-253 Bragança, Portugal; 3 Faculty of Physical Activity and Sports Sciences and Institute of Biomedicine (IBIOMED), Universidad De León, 24007 León, Spain; 4 Department of Physiotherapy, Federal University of Minas Gerais (UFMG), Belo Horizonte 31270-901, Brazil; leticiaamaral28@gmail.com; 5 Department of Sports, Higher Institute of Educational Sciences of the Douro, 4560-708 Penafiel, Portugal; 6 Department of Sports Sciences, Polytechnic of Guarda, 6300-559 Guarda, Portugal; zeteixeira1991@gmail.com; 7 Physiotherapy and Pain Group, Department of Physical Therapy, University of Alcala, 28801 Madrid, Spain

**Keywords:** postmenopausal women, physical activity, functional fitness, body composition, quality of life

## Abstract

Introduction: Postmenopausal aging is accompanied by declines in functional fitness, changes in body composition, and increased osteoporosis risk, which may affect quality of life. Understanding how these factors interrelate is important for supporting healthy aging. Objective: To examine the associations between accelerometer-measured physical activity, functional fitness, DEXA-derived body composition and bone parameters, and quality of life in postmenopausal women. Methods: Forty community-dwelling postmenopausal women (68.7 ± 5.7 years) participated in this cross-sectional study. Physical activity was assessed using a wrist-worn accelerometer for seven days. Body composition and bone health were measured by DEXA, functional fitness by the Senior Fitness Test, and quality of life by the WHOQOL-BREF. Spearman correlations were applied. Results: Associations were predominantly weak to moderate. Higher-intensity physical activity showed the strongest association with the Physical Health domain of quality of life (ρ ≈ 0.29). Total bone mineral density was also positively associated with perceived physical health (ρ ≈ 0.36). Adiposity was inversely related to light and lifestyle activity. Conclusion: Physical activity, functional fitness, body composition, and perceived physical health co-occur in postmenopausal women, supporting the relevance of promoting active lifestyles in this population.

## 1. Introduction

Postmenopausal aging is accompanied by accelerated biological degeneration largely driven by estrogen deficiency, which promotes increased bone resorption, reductions in lean mass, and redistribution of adipose tissue [1,2]. These changes contribute to a cascade of functional and metabolic impairments that increase the risk of osteoporosis and sarcopenia—two conditions that frequently coexist and potentiate each other [3,4]. Osteoporosis currently affects more than 200 million women worldwide and is projected to rise substantially in the coming decades due to increased life expectancy [5,6]. According to the International Osteoporosis Foundation, one in three women over the age of 50 will experience at least one fragility fracture, often associated with progressive loss of independence, increased frailty, chronic pain, and elevated mortality risk [7]. These consequences extend beyond physical impairment, frequently affecting psychosocial well-being, perceived competence, and overall quality of life [8,9,10].

In parallel with skeletal deterioration, postmenopausal women experience a gradual decline in functional fitness, including muscle strength, flexibility, balance, mobility, and aerobic capacity—key components for maintaining autonomy in activities of daily living [11,12,13]. Physical inactivity accelerates this decline, as older adults typically reduce time spent in moderate-to-vigorous physical activity while accumulating longer periods of sedentary behavior [14]. Conversely, women who maintain higher habitual physical activity levels or better functional performance generally exhibit more favorable body composition, improved bone integrity, and higher perceived quality of life, reflecting the multidimensional nature of active and healthy aging [15,16]. However, a substantial proportion of the existing evidence relies on self-reported physical activity measures, which are subject to recall bias and may inadequately capture free-living activity patterns and intensity distributions [17]. In addition, many studies examine physical activity, functional fitness, body composition, bone health, and quality of life in isolation rather than within an integrated framework, limiting a comprehensive understanding of how these constructs co-occur during aging [18,19].

The use of objective assessment methods—such as accelerometry for real-world physical activity monitoring and dual-energy X-ray absorptiometry (DEXA) for precise evaluation of body composition and bone parameters—offers a more robust approach to describing these interrelationships in postmenopausal women. Although several cross-sectional studies have examined associations among subsets of these variables, fewer investigations have simultaneously integrated objective physical activity assessment across intensity categories, functional fitness performance, DEXA-derived musculoskeletal indices, and multidomain quality of life measures in community-dwelling postmenopausal women who are not engaged in structured exercise programs [20,21,22]. As a result, uncertainty remains regarding which physical activity intensity bands and functional attributes are most consistently aligned with specific domains of quality of life, particularly those most directly related to physical function and health status.

Rather than proposing a novel causal model, an integrative and exploratory characterization of these domains may help clarify patterns of co-occurrence and inform the prioritization of targets for future hypothesis-driven and interventional research. In this context, the Physical Health domain of the WHOQOL-BREF represents the quality-of-life dimension most proximally related to physical function, musculoskeletal health, and daily autonomy, and may therefore be more sensitive to variations in physical activity, fitness, and body composition than psychological, social, or environmental domains.

Thus, the aim of this cross-sectional study was to describe the pattern and magnitude of associations between accelerometer-measured physical activity (across intensity categories and sedentary time), functional fitness performance, DEXA-derived body composition and bone indices, and quality of life in postmenopausal women, with particular emphasis on the Physical Health domain as the quality-of-life dimension most closely related to physical and musculoskeletal health.

## 2. Materials and Methods

### 2.1. Study Design and Participants

This cross-sectional study included postmenopausal women aged 55 years or older who were recruited from the local community. Eligible participants were required to be physically independent and free from acute musculoskeletal, cardiovascular, or neurological conditions that could compromise safe participation in functional assessments. Women undergoing pharmacological treatment specifically for osteoporosis or presenting unstable clinical conditions were excluded. All assessments were conducted during a single visit under standardized laboratory conditions.

Participant recruitment followed a non-probabilistic, convenience-based approach and was conducted through community health promotion initiatives and physical activity programs affiliated with the Polytechnic Institute of Bragança (IPB). The study included postmenopausal women aged 55 years or older, living independently in the community. To ensure safe participation in all assessments, eligible volunteers were required to be free from acute or unstable musculoskeletal, cardiovascular, neurological, or psychological conditions that could affect mobility or interfere with test execution. Additionally, all participants were required to present adequate visual and auditory acuity to understand instructions clearly and perform the tasks properly.

Exclusion criteria were defined to avoid factors that could influence functional performance or body composition results. Women were excluded if they (i) were currently enrolled in structured or supervised exercise programs; (ii) relied on mobility aids or demonstrated marked difficulty in performing activities of daily living; (iii) presented clinically relevant impairments affecting motor control, balance, or sensory perception; or (iv) were under pharmacological treatment known to directly affect bone metabolism or body composition (e.g., hormone replacement therapy or osteoporosis medication).

A total of 68 women were initially recruited through community-based health promotion initiatives. Of these, 46 met the eligibility criteria and agreed to participate. After baseline assessments, 40 women completed the functional fitness tests, DEXA scans, and quality-of-life questionnaires and were therefore included in the study sample.

Based on the predefined accelerometer wear-time criteria, valid accelerometer data were available for 31 participants. Consequently, analyses involving accelerometer-derived physical activity variables were conducted using this subsample, whereas all other analyses included the full sample of 40 women. This reduced sample size was taken into account in the interpretation of the accelerometer-based analyses, which were treated as exploratory.

The final sample had a mean age of 68.68 ± 5.74 years and a mean body mass of 70.47 ± 14.88 kg. All participants were biologically female and self-reported being postmenopausal. Participant characteristics are presented in Section 3.

### 2.2. Ethical Approval

This study was approved by the Ethics Committee of the Polytechnic Institute of Bragança under case number 2067313 and conducted in accordance with the ethical principles of the Declaration of Helsinki. All participants were informed about the study procedures and provided written informed consent prior to data collection.

### 2.3. Body Composition Assessment

Whole-body body composition and bone health were assessed using dual-energy X-ray absorptiometry (DEXA) [23], a gold-standard imaging method for evaluating musculoskeletal and metabolic health in older adults [24]. The scan provided quantitative measurements of total body mass, fat mass, lean soft tissue mass, bone mineral content (BMC), and bone mineral density (BMD). All assessments were conducted under standardized conditions in a controlled laboratory environment.

Participants were instructed to remove any metal accessories and to wear light, non-compressive clothing, being assessed barefoot to minimize measurement interference. Each volunteer was positioned in the standard anatomical supine position, aligned according to manufacturer specifications to ensure consistency across evaluations.

All measurements were conducted by a certified technician with prior experience in densitometry procedures, following the established operational guidelines for scan execution and quality control. Calibration of the DEXA device was verified daily before testing to guarantee accuracy and reliability of the measurements.

Bone mineral density (BMD), T-score, and Z-score values were obtained directly from the DEXA software output (enCORE™, GE Healthcare, version 18, Chicago, IL, USA), according to the manufacturer’s standard reference database. T-scores were calculated using a young adult female reference population, whereas Z-scores were calculated using an age- and sex-matched reference population, in accordance with World Health Organization (WHO) and International Society for Clinical Densitometry (ISCD) recommendations.

In the present study, total body BMD was used to provide a global indicator of skeletal status rather than to establish a clinical diagnosis of osteoporosis. Although site-specific measurements (e.g., lumbar spine or femoral neck) are recommended for diagnostic purposes, total body BMD is commonly applied in research settings to describe overall bone health and its relationship with functional and lifestyle-related factors.

Accordingly, BMD, T-score, and Z-score values were treated as descriptive research variables and were not used to classify participants diagnostically. Interpretation of these indices was therefore aligned with ISCD and WHO guidance, emphasizing their role in characterizing skeletal health rather than in defining osteoporosis or fracture risk.

### 2.4. Physical Fitness Assessment

Functional fitness was evaluated using the Senior Fitness Test battery developed by Rikli and Jones [25], which is widely recognized as a reliable and valid tool for assessing physical function in older adults [25]. This protocol examines multiple components of health-related fitness, including muscular strength, flexibility, agility, balance, and aerobic endurance, through six standardized field tests.

Lower limb strength was assessed using the 30-s Chair Stand Test, where participants performed the maximum number of sit-to-stand repetitions within 30 s. Upper limb strength was measured with the Arm Curl Test, consisting of the number of curls executed in 30 s using a 2 kg dumbbell. Flexibility of the lower extremities was measured through the Chair Sit-and-Reach Test, in which the distance (in centimeters) between the extended fingertips and the toes was recorded. Upper body flexibility was assessed using the Back Scratch Test, quantifying the distance between the middle fingers of both hands.

Functional mobility and agility were examined using the Timed Up and Go (TUG) test, which measures the time (in seconds) required to stand up from a chair, walk 3 m, turn, return, and sit down. Finally, aerobic endurance was evaluated using the 2-min Step Test, where participants were instructed to march in place and the number of knee lifts reaching hip height completed within 2 min was recorded.

This testing protocol is extensively adopted in studies involving older adults and is considered appropriate for detecting functional performance differences and age-related physical decline.

### 2.5. Quality of Life Assessment

Quality of life was assessed using the WHOQOL-BREF [26], a validated instrument developed by the World Health Organization to evaluate health-related quality of life across four domains: Physical Health (items 3, 4, 10, 15, 16, 17, 18), Psychological (items 5, 6, 7, 11, 19, 26), Social Relationships (items 20, 21, 22), and Environment (items 8, 9, 12, 13, 14, 23, 24, 25). The questionnaire consists of 26 items, scored on a 5-point Likert scale, with higher scores indicating better perceived quality of life.

The instrument was administered in paper-and-pen format, and all participants completed it individually in a quiet room, without external interference. They were instructed to respond independently, with no influence or assistance from other participants, and were only allowed to ask clarifying questions in case of doubt. A qualified researcher was present throughout administration to ensure standardized procedures and to provide neutral clarification when necessary, without influencing the content of responses.

Domain scores were calculated in accordance with the official WHOQOL-BREF scoring guidelines, converted to a 0–100 scale, with higher values representing better quality of life.

### 2.6. Accelerometer-Based Physical Activity Assessment

Habitual physical activity was objectively assessed using a triaxial accelerometer (ActiGraph GT3X, ActiGraph LLC, Pensacola, FL, USA) [27]. The device was worn on the right wrist, secured with an adjustable band, during waking hours for seven consecutive days, except during water-based activities (e.g., bathing or swimming). Wrist placement was selected to maximize wear compliance and data completeness, as this positioning has been shown to be better tolerated in older adult populations compared with hip-worn protocols.

We acknowledge that wrist placement may influence activity classification compared with hip-worn devices, particularly by capturing upper-limb movements in addition to ambulatory activity. For this reason, physical activity intensity was derived using cut-points specifically validated for wrist-worn ActiGraph devices in older adults.

Accelerometer data were processed to quantify the mean daily accumulated time spent in sedentary behavior and across five physical activity intensity categories: lifestyle, light, moderate, vigorous, and very vigorous physical activity. Intensity thresholds were defined according to the wrist-specific cut-points proposed and validated by Bammann et al. [27]. Within this framework, “lifestyle physical activity” was operationally defined as low-intensity ambulatory and non-ambulatory movement exceeding sedentary behavior but not reaching the threshold for light physical activity, reflecting common activities of daily living such as household tasks and slow walking.

A day was considered valid if the accelerometer was worn for at least 10 h, and only participants with a minimum of four valid days, including at least one weekend day, were included in the analyses, in accordance with established guidelines [28]. Non-wear time was identified using standard algorithms based on continuous intervals of zero-count epochs. Based on these criteria, valid accelerometer data were available for 31 participants. Consequently, analyses involving accelerometer-derived variables were conducted using this subsample and interpreted descriptively.

Accelerometry is widely recognized as a robust method for objectively quantifying free-living physical activity in older adults; however, interpretation of wrist-derived intensity categories should consider the influence of device placement when comparing results across studies using different accelerometer protocols [29].

### 2.7. Statistical Analysis

Descriptive statistics were calculated for all variables and are presented as mean, standard deviation, median, minimum, and maximum values. Data distribution normality was assessed using the Shapiro–Wilk test [30], which is recommended for evaluating normality in small to moderate sample sizes. Inspection of the normality results indicated that several variables deviated from a normal distribution. Based on these findings, and considering the sample size, non-parametric statistical procedures were adopted throughout the analyses.

Spearman’s rank correlation coefficient (ρ) was used to examine the associations between quality-of-life domains [31], DEXA-derived body composition and bone health indicators, accelerometer-measured physical activity levels, and functional fitness performance. The magnitude of the correlations was interpreted according to conventional thresholds: negligible (<0.10), weak (0.10–0.29), moderate (0.30–0.49), strong (0.50–0.69), and very strong (≥0.70) [32]. Given the exploratory nature of the study and the limited sample size, no formal correction for multiple testing was applied. We acknowledge that examining a large number of correlations increases the probability of type I error; therefore, the results are interpreted descriptively, with emphasis on the direction, magnitude, and precision of associations rather than on isolated p-values.

Ninety-five percent confidence intervals (95% CI) for all Spearman correlation coefficients were estimated using a bootstrap resampling procedure to provide measures of precision, particularly in the context of small sample size and multiple comparisons.

Missing data were examined prior to analysis. For accelerometer-derived variables, only participants meeting the predefined wear-time criteria (≥10 h/day, ≥4 valid days including at least one weekend day) were included in the analyses. No data imputation procedures were applied, and all analyses were conducted using available-case data.

An a priori sample size calculation was not performed due to the exploratory design of the study, which aimed to describe patterns of co-occurrence rather than to test predefined confirmatory hypotheses.

All statistical analyses were performed using RStudio (version 2024.09.0+375), employing the packages psych, Hmisc, janitor, corrplot, and writexl. Statistical significance was set at *p* < 0.05.

Detailed results of the Shapiro–Wilk normality tests are provided in the Supplementary Material (Table S1).

## 3. Results

Table 1 presents the demographic and anthropometric characteristics of the study participants.

Descriptive statistics for body composition, bone parameters, functional fitness, accelerometer-measured physical activity, and quality-of-life domains are summarized in Table 2, Table 3, Table 4 and Table 5. Several variables showed asymmetric distributions, supporting the use of non-parametric analyses, as described in the Statistical Analysis section. Detailed results of normality testing are provided in the Supplementary Materials (Table S1).

Functional fitness performance is described in Table 3, which includes measures of muscular strength, flexibility, mobility, and aerobic endurance. While most functional tests showed approximately symmetric distributions, flexibility- and mobility-related outcomes (Chair Sit-and-Reach, Back Scratch, and Timed Up and Go) exhibited significant deviations from normality.

Table 4 summarizes accelerometer-derived physical activity patterns based on valid wear-time criteria (n = 31). Participants accumulated the greatest proportion of daily time in sedentary behavior, followed by lifestyle and light physical activity, whereas time spent in moderate, vigorous, and very vigorous intensities was comparatively low. Several physical activity intensity variables displayed non-normal distributions, supporting the use of non-parametric analyses.

Quality-of-life domain scores assessed by the WHOQOL-BREF are presented in Table 5. Mean scores were highest for the Environment and Social Relationships domains, whereas greater variability was observed in the Physical Health domain.

Given the presence of non-normal distributions across multiple variables, all associations were examined using Spearman’s rank correlation coefficients (ρ). Correlation analyses explored the relationships between quality of life, physical activity, functional fitness, and body composition variables. Ninety-five percent confidence intervals (95% CI) for all correlation coefficients were estimated using a bootstrap resampling procedure to provide measures of precision and are reported in the Supplementary Material (Table S2).

**Figure 1:**
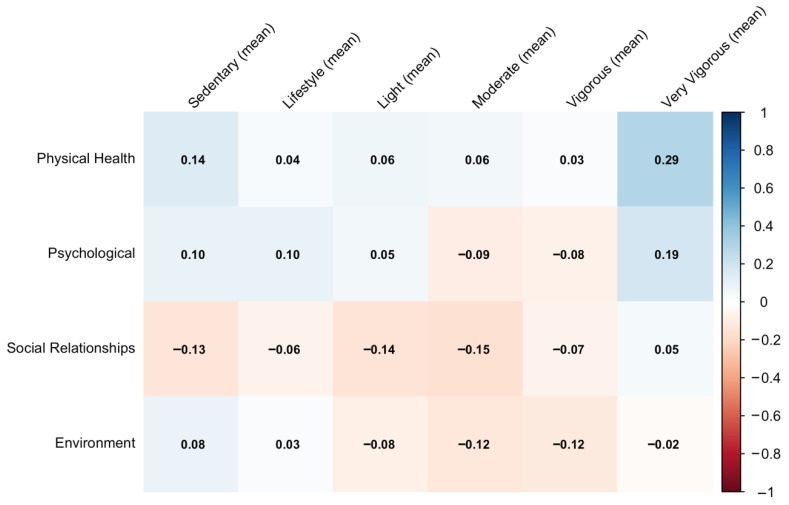
Correlation between quality of life domains and accelerometer-measured physical activity.

Figure 1 presents the correlations between quality of life domains and accelerometer-derived physical activity variables. Overall, the correlations are weak across domains; however, Very Vigorous (mean) shows the strongest positive association with Physical Health (ρ = 0.29). Lifestyle, light, moderate and vigorous activity show only minimal correlations with the Psychological, Social Relationships, and Environment domains, all remaining near null.

**Figure 2:**
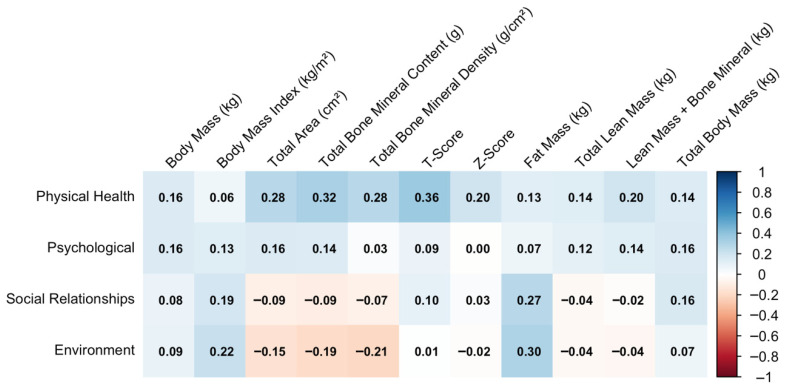
Correlation between quality-of-life domains and DEXA-derived bone and body composition variables.

Figure 2 displays the correlations between quality of life domains and DEXA-derived body composition and bone parameters. The Physical Health domain shows the highest positive correlations, particularly with Total Bone Mineral Density (g/cm^2^) (ρ = 0.36), followed by Total Bone Mineral Content, Total Area, and Body Mass Index (ρ ≈ 0.28–0.32). The Psychological domain follows a similar but weaker pattern, while Social Relationships and Environment domains generally show negligible to small correlations, with no clear directional trends.

**Figure 3:**
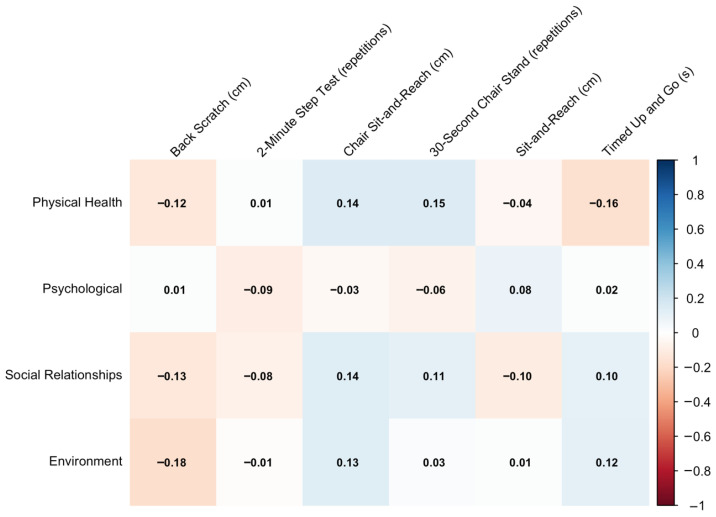
Correlation between quality-of-life domains and functional fitness performance.

Figure 3 shows the correlations between quality of life and functional fitness performance. Again, correlations are mostly weak, although Chair Sit-and-Reach and 30-s Chair Stand tests display modest positive correlations with Physical Health (ρ = 0.14–0.15). The Timed Up and Go test shows a weak negative correlation with Physical Health (ρ = −0.16), consistent with its inverse performance scale. The remaining associations across domains remain close to zero.

**Figure 4:**
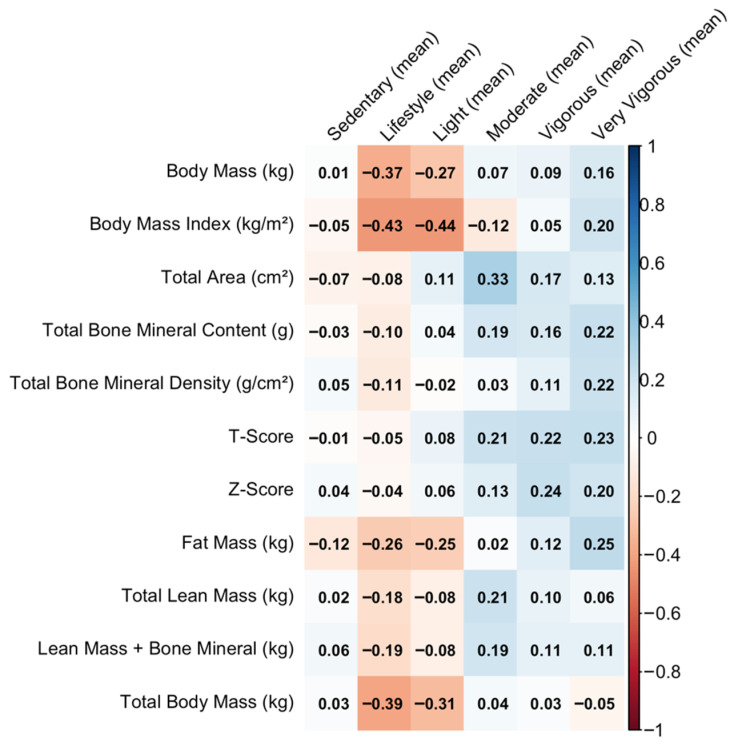
Correlation between DEXA-derived body composition and bone variables and accelerometer-measured physical activity.

Figure 4 illustrates the correlations between DEXA variables and accelerometer-derived physical activity. The strongest relationships are observed between adiposity markers and Lifestyle, Light, and Sedentary physical activity. Body Mass Index, Fat Mass, and Total Body Mass show moderate negative correlations with Lifestyle and Light activity (ρ = −0.25 to −0.44). Conversely, Very Vigorous activity shows weak-to-moderate positive correlations with bone and lean tissue indicators (ρ up to 0.22).

**Figure 5:**
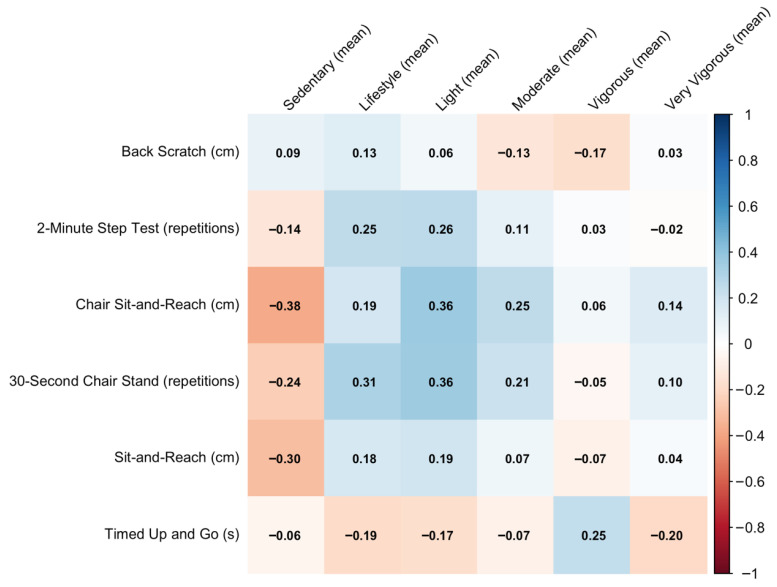
Correlation between functional fitness performance and accelerometer-measured physical activity.

Figure 5 presents the correlations between functional fitness measures and accelerometry. The Chair Sit-and-Reach and 2-min Step Test show the most consistent positive associations, particularly with Lifestyle and Light activity (ρ = 0.19–0.36). Sedentary time tends to correlate negatively with performance-based fitness outcomes—especially flexibility (ρ = −0.38 in Sit-and-Reach). Very Vigorous activity shows only small or near-null correlations with functional outcomes.

**Figure 6:**
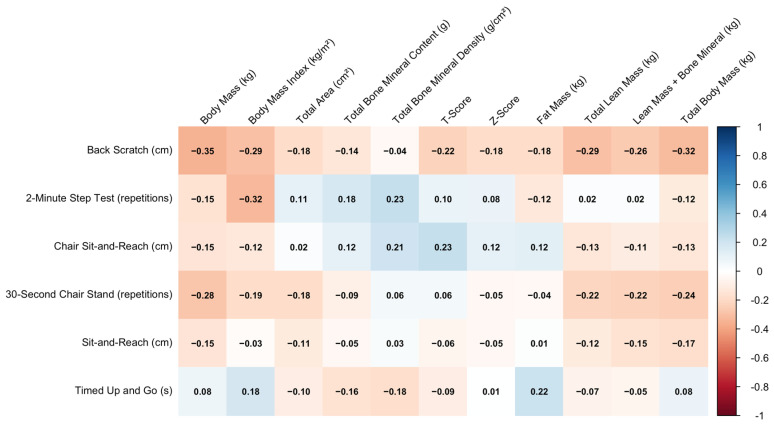
Correlation between functional fitness performance and DEXA-derived body composition.

Finally, Figure 6 displays the correlations between functional fitness and DEXA variables. Performance in the 30-s Chair Stand test demonstrates weak-to-moderate negative correlations with adiposity indicators (ρ = −0.22 to −0.24), while BMD and BMC show weak positive correlations with functional performance—especially Chair Sit-and-Reach and 2-Minute Step Test. As expected, Timed Up and Go shows inverse relationships, with slower times modestly linked to higher adiposity.

## 4. Discussion

This study sought to describe the pattern of associations between objectively measured physical activity, functional fitness, body composition, and quality of life in postmenopausal women using an integrative and exploratory approach. Rather than proposing novel causal relationships, the primary contribution of this work lies in the simultaneous examination of these domains using objective assessment tools, allowing a coherent characterization of how physical activity intensities, musculoskeletal profiles, and functional performance co-occur with different dimensions of quality of life in community-dwelling women not engaged in structured exercise programs. As expected for multifactorial aging-related constructs assessed under free-living conditions, the observed associations were predominantly weak to moderate in magnitude.

Within this integrated framework, the Physical Health domain of the WHOQOL-BREF emerged as the quality-of-life dimension most consistently aligned with physical activity, functional fitness, and body composition variables. In particular, higher-intensity physical activity showed small but comparatively stronger associations with perceived physical health than lower-intensity activity or sedentary time. These findings suggest that, among the quality-of-life domains, Physical Health may be more proximally related to physical and musculoskeletal characteristics, whereas other domains are likely influenced by a broader set of determinants beyond physical activity alone.

For interpretative purposes, the Physical Health domain of the WHOQOL-BREF was therefore considered the primary outcome, while the remaining quality-of-life domains were included to provide contextual information rather than being weighted equally.

In contrast, associations between physical activity and the psychological, social, and environmental domains of quality of life were generally weak or near null. This pattern reinforces the multidimensional nature of quality of life, which is shaped not only by physical activity and functional capacity, but also by pain, emotional health, social participation, environmental context, and menopausal-related symptoms [33,34,35]. Chronic pain, psychological distress, and menopausal symptoms have all been shown to exert independent and substantial effects on perceived quality of life, potentially attenuating bivariate associations with physical activity when examined in isolation.

Consistent with previous literature [33,36,37,38], greater engagement in physical activity was associated with more favorable body composition and bone-related indicators. However, these associations should be interpreted descriptively rather than causally, reflecting patterns of co-occurrence rather than evidence of direct effects. In the present sample, total bone mineral density, bone mineral content, and body mass index displayed small positive associations with the Physical Health domain of quality of life, suggesting that women with more favorable musculoskeletal profiles tend to report better perceived physical health.

Previous interventional studies have demonstrated that resistance and aerobic exercise programs can lead to improvements or attenuation of age-related declines in bone mineral density [37]. In the present cross-sectional study, however, the observed associations between bone-related indicators and physical activity or quality-of-life domains should be interpreted as descriptive patterns of co-occurrence rather than evidence of training effects.

Within the limitations of a cross-sectional design, the present findings suggest that higher levels of habitual physical activity tend to co-occur with more favorable body composition and functional profiles, which are, in turn, aligned with higher scores in the Physical Health domain of quality of life. However, these associations do not imply directionality or causality and should be interpreted cautiously. This idea is reinforced when we analyze the results of our study, which showed that, with regard to body composition, especially total bone mineral density, bone mineral content, and body mass index, in the physical health domain there is a positive correlation, indicating that the better the bone health and BMI, the better the perception of physical health of postmenopausal women. In relation to the psychological, social relations, and environmental domains, these show weak correlations, highlighting the idea that quality of life is influenced by multiple factors [35].

Sedentary participants or those with low levels of light movement demonstrated higher fat mass, whereas moderate and vigorous physical activity correlated more strongly with bone indicators. This relationship is physiologically plausible, as higher-intensity mechanical loading provides greater osteogenic stimulus and promotes musculoskeletal adaptation, particularly in postmenopausal women [39].

Functional fitness also showed weak associations with quality of life, particularly within the Physical Health domain. The sit-and-reach and 30 s chair stand tests demonstrated positive correlations with the Physical Health domain, reinforcing that flexibility and muscular strength contribute to autonomy in activities of daily living, independence, and better perceived physical well-being [40]. Conversely, the negative correlation found between TUG performance and physical health was expected, as longer times reflect lower functional capacity and poorer physical quality-of-life perception.

As in other domains, correlations with psychological, social, and environmental quality of life were weak, further confirming its multidimensional nature [36].

Weak-to-moderate negative correlations were observed between fat mass and performance on the 30 s chair stand test, suggesting that excess adiposity may impair mobility and increase the metabolic cost of movement. In contrast, weak but positive correlations between BMD/BMC and functional performance indicate that women with better neuromuscular fitness tend to exhibit healthier bone profiles.

Given the accelerated bone loss associated with declining estrogen levels in postmenopausal women, the patterns observed in this study are consistent with existing evidence highlighting the relevance of muscular strength, mobility, and habitual physical activity in musculoskeletal health across aging [41].

Taken together, the findings indicate that physical activity, functional fitness, and body composition are interrelated with certain aspects of quality of life in postmenopausal women, particularly within the Physical Health domain. Although the associations observed were predominantly weak, their consistency supports the relevance of considering these constructs jointly in aging research. This interpretation is consistent with prior literature [39,42,43] and World Health Organization guidance, which suggest that even small increases in daily movement may be associated with benefits to physical and mental health [40].

From a public health perspective, these results are aligned with existing recommendations that encourage regular physical activity and reduced sedentary behavior in older adults. However, longitudinal and interventional studies are required to determine whether changes in physical activity lead to clinically meaningful improvements in musculoskeletal health or quality of life. This study has several limitations. The small sample size limits generalizability, and the cross-sectional design does not allow causal inference or analysis of temporal changes. Quality of life may also have been influenced by unmeasured external factors (e.g., mood, pain levels, socioeconomic conditions, dietary habits), which may have introduced confounding effects. In addition, the reliance on bivariate correlations represents a limitation, as potential confounders such as age, years since menopause, body mass index, and sedentary time were not statistically controlled for. These factors may influence the observed associations and should be addressed in future multivariable, longitudinal, or interventional studies with adequate statistical power. As participants were recruited using convenience sampling from community health initiatives, the sample may not be fully representative of the broader population of postmenopausal women. It should be noted that much of the existing evidence in this field is derived from cross-sectional studies, which limits causal inference and may partly explain the modest associations reported across studies.

Future research should prioritize randomized controlled trials and longitudinal designs to clarify causal relationships over time and include larger, more diverse samples to improve external validity.

## 5. Conclusions

These findings support public health strategies that encourage regular engagement in moderate-to-vigorous physical activity and the maintenance of functional fitness to support physical health and musculoskeletal integrity in postmenopausal women.

## Figures and Tables

**Table 1:** Participant characteristics (n = 40).

Characteristic	N	Mean	SD	Median	Min.	Max.
Age (years)	40	68.68	5.74	69.00	56.00	80.00
Height (cm)	40	157.62	6.21	157.00	145.00	171.00
Body mass (kg)	40	70.47	14.88	69.30	46.20	108.40
Body mass index (kg/m^2^)	40	28.42	5.73	27.80	18.90	41.60
Years since menopause	40	18.91	7.22	18.00	5.00	34.00

Note: SD (Standard Deviation). Values are presented as mean ± SD and median (min–max).

**Table 2:** Descriptive statistics of body composition and bone variables (DEXA).

Variable	Mean ± SD	Median (Min–Max)
Total body area (cm^2^)	18.421.6 ± 1.942.3	18.307.0 (14.820.0–22.965.0)
Bone mineral density—Total (g/cm^2^)	1.03 ± 0.11	1.02 (0.79–1.29)
Bone mineral content—Total (kg)	2.12 ± 0.38	2.09 (1.45–2.98)
T-score (Total body)	−0.87 ± 1.12	−0.90 (−3.10–1.40)
Z-score (Total body)	0.21 ± 0.94	0.18 (−2.10–2.30)
Fat mass (kg)	30.61 ± 9.92	29.40 (13.20–55.60)
Total lean mass (kg)	38.07 ± 5.68	37.50 (27.90–50.40)
Lean mass + bone mineral (kg)	40.19 ± 5.93	39.60 (29.60–53.10)

Note: SD (Standard Deviation).

**Table 3:** Descriptive statistics of functional fitness variables.

Variable	Mean ± SD	Median (Min–Max)
30 s Chair Stand (reps)	14.35 ± 3.47	14.00 (7.00–22.00)
Arm Curl (reps)	15.92 ± 3.86	16.00 (8.00–24.00)
Chair Sit-and-Reach (cm)	−1.84 ± 7.52	−2.00 (−18.00–14.00)
Back Scratch (cm)	−6.41 ± 8.29	−6.00 (−26.00–8.00)
Timed Up and Go (s)	7.21 ± 1.31	7.00 (5.10–11.20)
2-min Step Test (reps)	93.45 ± 18.76	92.00 (55.00–132.00)

Note: SD (Standard Deviation).

**Table 4:** Accelerometer-measured physical activity (valid data only, n = 31).

Variable (min/day)	Mean ± SD	Median (Min–Max)
Sedentary time	643.28 ± 92.47	651.00 (455.00–790.00)
Lifestyle activity	152.41 ± 46.38	148.00 (74.00–261.00)
Light activity	78.62 ± 31.54	74.00 (29.00–165.00)
Moderate activity	22.14 ± 12.27	20.00 (5.00–52.00)
Vigorous activity	6.43 ± 4.19	5.00 (1.00–18.00)
Very vigorous activity	2.11 ± 1.83	2.00 (0.00–7.00)

Note: SD (Standard Deviation); Accelerometer data include only participants with ≥4 valid days (≥10 h/day), including ≥1 weekend day.

**Table 5:** WHOQOL-BREF domain scores.

Domain	Mean ± SD	Median (Min–Max)
Physical Health	64.32 ± 12.41	65.00 (38.00–88.00)
Psychological	68.91 ± 11.23	69.00 (41.00–88.00)
Social Relationships	71.44 ± 14.87	75.00 (33.00–100.00)
Environment	73.86 ± 10.18	75.00 (47.00–94.00)

Note: SD (Standard Deviation); Values are presented as mean ± SD and median (min–max).

## Data Availability

The data presented in this study are not publicly available due to ethical and privacy restrictions, as they contain sensitive information from human participants. Data are available from the corresponding author upon reasonable request and subject to approval by the institutional ethics committee.

## Supplementary Materials

The following supporting information can be downloaded at: https://www.mdpi.com/article/10.3390/sports14020054/s1, Table S1: Results of Shapiro–Wilk normality tests for study variables; Table S2: Spearman correlations with 95% CI.
