# Supplementary material for: Associations Between Quality of Life, Functional Fitness, Body Composition, and Accelerometer-Measured Physical Activity in Postmenopausal Women: A Cross-Sectional Study
Source: Sports (Basel). 2026 Feb 3;14(2):54. doi: 10.3390/sports14020054 (PMC12944340; doi:10.3390/sports14020054)
Supplement: Supplementary file 1 [file sports-14-00054-s001.zip › Supplementary Table S1.pdf]

Table S1. Results of Shapiro–Wilk normality tests for study variables.

**Variable**	**N**	**Shapiro–Wilk W**	**p-value**	**Distribution**
Age (years)	40	0.973	0.214	Normal
Height (cm)	40	0.965	0.083	Normal
Body mass (kg)	40	0.942	0.041	Non-normal
Body mass index (kg/m^2)	40	0.938	0.032	Non-normal
Years since menopause	40	0.969	0.118	Normal
Total body BMD (g/cm^2)	40	0.951	0.067	Normal
Bone mineral content (kg)	40	0.947	0.054	Normal
Fat mass (kg)	40	0.926	0.021	Non-normal
Lean mass (kg)	40	0.961	0.097	Normal
30-s Chair Stand (reps)	40	0.931	0.028	Non-normal
TUG (s)	40	0.918	0.014	Non-normal
WHOQOL Physical Health	40	0.958	0.082	Normal
WHOQOL Psychological	40	0.962	0.094	Normal
WHOQOL Social	40	0.949	0.061	Normal
WHOQOL Environment	40	0.955	0.075	Normal

Note: Normality was assessed using the Shapiro–Wilk test. Variables with $p < 0.05$ were considered non-normally distributed. Abbreviations: BMD, bone mineral density; TUG, Timed Up and Go.
