# Supplementary material for: Associations Between Quality of Life, Functional Fitness, Body Composition, and Accelerometer-Measured Physical Activity in Postmenopausal Women: A Cross-Sectional Study
Source: Sports (Basel). 2026 Feb 3;14(2):54. doi: 10.3390/sports14020054 (PMC12944340; doi:10.3390/sports14020054)
Supplement: Supplementary file 1 [file sports-14-00054-s001.zip › Supplementary Table S2.pdf]

1. Table S2 – Spearman correlations with 95% CI

Variable_1	Variable_2	n	Spearman ρ	95% CI	p-value
lean_total_kg	lean_bmc_total_kg	80	0.973	[0.945, 0.986]	0.00000000000000000000000000000000000000000000000000129
mas_corp_pre	total_mass_total_kg	80	0.943	[0.822, 0.996]	0.00000000000000000000000000000000000000378000000000000
area_total_1	bmc_total_1	80	0.930	[0.879, 0.961]	0.00000000000000000000000000000000001179999999999999857
bmc_total_1	bmd_total_1	80	0.890	[0.812, 0.938]	0.00000000000000000000000000237000000000000001414553783
mas_corp_pre	lean_bmc_total_kg	80	0.875	[0.802, 0.919]	0.00000000000000000000000002900000000000000398621047766
mas_corp_pre	lean_total_kg	80	0.872	[0.797, 0.917]	0.00000000000000000000000005729999999999999847493900521
area_total_1	lean_bmc_total_kg	80	0.869	[0.777, 0.923]	0.00000000000000000000000014699999999999999704916808832
lean_total_kg	total_mass_total_kg	80	0.837	[0.721, 0.907]	0.00000000000000000000036599999999999998346198435951414
t_score_1	z_score_1	80	0.835	[0.654, 0.955]	0.00000000000000000000059900000000000001288764578026838
lean_bmc_total_kg	total_mass_total_kg	80	0.835	[0.72, 0.905]	0.00000000000000000000062900000000000003080246992157754
area_total_1	lean_total_kg	80	0.831	[0.725, 0.894]	0.00000000000000000000143000000000000003893053416561687
mas_corp_pre	imc1	80	0.816	[0.707, 0.886]	0.00000000000000000003079999999999999933387104374537385
bmc_total_1	lean_bmc_total_kg	80	0.779	[0.658, 0.863]	0.00000000000000001819999999999999868280739342747826590
imc1	total_mass_total_kg	80	0.760	[0.602, 0.864]	0.00000000000000030299999999999999317359131161935250086
bmc_total_1	lean_total_kg	80	0.722	[0.585, 0.823]	0.00000000000004069999999999999949440656570240102983456
mas_corp_pre	area_total_1	80	0.710	[0.579, 0.802]	0.00000000000015800000000000000833314865440739149259389

Variable_1	Variable_2	n	Spearman ρ	95% CI	p-value
area_total_1	bmd_total_1	80	0.684	[0.525, 0.812]	0.000000000002659999999999999747712575369409998284892
area_total_1	total_mass_total_kg	80	0.680	[0.53, 0.792]	0.00000000000386000000000000011219394808131095617187434
lifestyle_mean	light_mean	60	0.741	[0.596, 0.839]	0.00000000001240000000000000047760864085445411042138431
flex_ant_1	lev_set_cad_1	80	0.666	[0.538, 0.77]	0.00000000001619999999999999963377401324662516451241556
physical_health_1	psychological_1	80	0.636	[0.492, 0.751]	0.000000000022900000000000001054017168734296258300608962
mas_corp_pre	bmc_total_1	80	0.611	[0.456, 0.735]	0.000000000177000000000000009162685309782722317684111601
bmc_total_1	t_score_1	80	0.601	[0.416, 0.746]	0.0000000003760000000000000025072239613231281363248115213
bmc_total_1	total_mass_total_kg	80	0.577	[0.397, 0.716]	0.0000000021200000000000000123765167764963018859347698708
bmd_total_1	t_score_1	80	0.546	[0.343, 0.713]	0.00000016099999999999999906719310837494019139626288961
bmd_total_1	z_score_1	80	0.545	[0.338, 0.707]	0.00000016799999999999999557405633895251773068935108313
imc1	lean_total_kg	80	0.541	[0.336, 0.71]	0.00000021699999999999999759187855469244610162604658399
bmd_total_1	lean_bmc_total_kg	80	0.534	[0.325, 0.711]	0.00000034299999999999998765497590848261300067179035977
imc1	mas_gorda_kg	80	0.533	[0.338, 0.683]	0.00000036199999999999998573639884910657649186305206968
imc1	lean_bmc_total_kg	80	0.523	[0.319, 0.688]	0.00000063000000000000000325504597234460568699887517141
area_total_1	t_score_1	80	0.522	[0.322, 0.677]	0.00000069599999999999998544534477590284282655375136528
bmc_total_1	z_score_1	80	0.520	[0.317, 0.688]	0.000000757000000000000001550750630338604274527369852876
light_mean	moderate_mean	60	0.573	[0.365, 0.725]	0.000001720000000000000004922169442866941579950434970669
bmd_total_1	lean_total_kg	80	0.470	[0.267, 0.656]	0.00001079999999999999993479608134672176333879178855568

Variable_1	Variable_2	n	Spearman ρ	95% CI	p-value
lev_set_cad_1	sent_cam_sent_1	80	-0.461	[-0.632, -0.256]	0.000016900000000000008234298659592909075399802532047 0
social_relantionships_1	environment_1	80	0.461	[0.251, 0.63]	0.0000169999999999999986599261148079165195667883381248
min_step_1	flex_ant_1	80	0.457	[0.28, 0.619]	0.0000204000000000000011944438493838305248573306016624 0
moderate_mean	vigorous_mean	60	0.514	[0.283, 0.699]	0.0000261999999999999998731917139060954013984883204102 5
min_step_1	lev_set_cad_1	80	0.419	[0.221, 0.601]	0.0001079999999999999959598290244500162771146278828382 5
area_total_1	z_score_1	80	0.408	[0.203, 0.592]	0.000174999999999999998221908437123772728222777740657330
mas_corp_pre	mas_gorda_kg	80	0.405	[0.204, 0.582]	0.000196999999999999990873619792886017876298865303397 18
mas_corp_pre	bmd_total_1	80	0.387	[0.168, 0.585]	0.000395999999999999997615102165227085606602486222982407
t_score_1	lean_bmc_total_kg	80	0.385	[0.165, 0.57]	0.000427000000000000002493491524369062517507700249552727
sedentary_mean	moderate_mean	60	-0.438	[-0.638, -0.197]	0.000465999999999999997123828476830453837465029209852 22
physical_health_1	environment_1	80	-0.382	[-0.583, -0.129]	0.000471999999999999998033517467632691477774642407894135
imc1	light_mean	60	-0.437	[-0.613, -0.227]	0.000485999999999999999537175776609387867210898548364639
flex_ant_1	sent_cam_sent_1	80	-0.381	[-0.551, -0.176]	0.000497000000000000004590772206825022294651716947555542
imc1	lifestyle_mean	60	-0.434	[-0.629, -0.211]	0.000524000000000000005167394290239712972834240645170212
flex_ant_1	sent_alc_1	80	0.366	[0.148, 0.55]	0.000832999999999999997310484722845558280823752284049988
physical_health_1	t_score_1	80	0.364	[0.149, 0.548]	0.000889000000000000003325117958752343838568776845932007
bmd_total_1	total_mass_total_kg	80	0.361	[0.142, 0.569]	0.001000000000000000020816681711721685132943093776702 88

Variable_1	Variable_2	n	Spearman ρ	95% CI	p-value
sedentary_mean	vigorous_mean	60	-0.404	[-0.602, -0.161]	0.0013600000000000009769962616701377555727958679199219
mas_corp_pre	alc_atr_cos_1	80	-0.345	[-0.542, -0.122]	0.00169999999999999990528409821166633264510892331600189
lev_set_cad_1	sent_alc_1	80	0.344	[0.139, 0.516]	0.00177000000000000008888723090905159551766701042652130
mas_corp_pre	t_score_1	80	0.341	[0.136, 0.527]	0.00199000000000000001540434446667404699837788939476013
total_mass_total_k g	lifestyle_mean	60	-0.391	[-0.579, -0.161]	0.00202000000000000009409140133698201680090278387069702
mas_gorda_kg	total_mass_total_kg	80	0.336	[0.125, 0.522]	0.00227999999999999990868415622458087455015629529953003
t_score_1	lean_total_kg	80	0.332	[0.109, 0.528]	0.00263999999999999998556710067987296497449278831481934
sedentary_mean	flex_ant_1	60	-0.378	[-0.573, -0.138]	0.002890000000000000020761170560490427305921912193298340
physical_health_1	bmc_total_1	80	0.324	[0.127, 0.496]	0.003389999999999999978433917746656334202270954847335815
imc1	min_step_1	80	-0.323	[-0.51, -0.124]	0.003429999999999999988925525329364063509274274110794067
mas_corp_pre	lifestyle_mean	60	-0.368	[-0.557, -0.132]	0.003830000000000000007105427357601001858711242675781250
total_mass_total_k g	alc_atr_cos_1	80	-0.316	[-0.517, -0.098]	0.004250000000000000030531133177191804861649870872497559
light_mean	flex_ant_1	60	0.363	[0.114, 0.575]	0.004420000000000000031752378504279477056115865707397461
light_mean	lev_set_cad_1	60	0.356	[0.103, 0.556]	0.005270000000000000037858605139717838028445839881896973
t_score_1	total_mass_total_kg	80	0.305	[0.083, 0.495]	0.00591999999999999991506793861617552465759217739105225
environment_1	mas_gorda_kg	80	0.304	[0.091, 0.499]	0.006190000000000000018957058145474547927733510732650757
imc1	alc_atr_cos_1	80	-0.294	[-0.511, -0.059]	0.008019999999999999935162975361890858039259910583496094

Variable_1	Variable_2	n	Spearman ρ	95% CI	p-value
lean_total_kg	alc_atr_cos_1	80	-0.294	[-0.502, -0.053]	0.0080599999999999945654582944598587346263229846954346
area_total_1	moderate_mean	60	0.328	[0.075, 0.54]	0.0106000000000000004718447854656915296800434589385986
psychological_1	environment_1	80	-0.281	[-0.469, -0.058]	0.0115999999999999920063942226988729089498519897460938
physical_health_1	area_total_1	80	0.279	[0.078, 0.463]	0.01230000000000000016930901125533637241460382938385010
mas_corp_pre	lev_set_cad_1	80	-0.279	[-0.485, -0.056]	0.01230000000000000016930901125533637241460382938385010
imc1	area_total_1	80	0.278	[0.064, 0.477]	0.01239999999999999956423746283462605788372457027435303
physical_health_1	bmd_total_1	80	0.278	[0.061, 0.473]	0.01260000000000000008881784197001252323389053344726562
social_relantionships_1	mas_gorda_kg	80	0.273	[0.044, 0.479]	0.014200000000000000081601392309949005721136927604675293
lifestyle_mean	lev_set_cad_1	60	0.314	[0.059, 0.531]	0.01469999999999999952537965697274557896889746189117432
total_mass_total_kg	light_mean	60	-0.312	[-0.518, -0.054]	0.01510000000000000057454041524351850966922938823699951
lean_bmc_total_kg	alc_atr_cos_1	80	-0.262	[-0.471, -0.023]	0.018700000000000000134336985979643941391259431838989258
sedentary_mean	sent_alc_1	60	-0.301	[-0.533, -0.035]	0.0194999999999999997224442438437108648940920829772949
physical_health_1	veryvig_mean	60	0.290	[0.085, 0.477]	0.024400000000000000154876111935209337389096617698669434
vigorous_mean	veryvig_mean	60	0.282	[0.096, 0.452]	0.02879999999999999921174165251613885629922151565551758
total_mass_total_kg	lev_set_cad_1	80	-0.243	[-0.455, -0.013]	0.029600000000000000131006316905768471769988536834716797
mas_corp_pre	light_mean	60	-0.274	[-0.489, -0.015]	0.03440000000000000002220446049250313080847263336181641

Variable_1	Variable_2	n	Spearman ρ	95% CI	p-value
bmd_total_1	min_step_1	80	0.233	[0.012, 0.442]	0.03740000000000000268673971959287882782518863677978516
t_score_1	mas_gorda_kg	80	0.232	[-0.014, 0.443]	0.03860000000000000236477504245158343110233545303344727
t_score_1	flex_ant_1	80	0.231	[0.016, 0.417]	0.03889999999999999708011344523583829868584871292114258
light_mean	min_step_1	60	0.264	[0.012, 0.506]	0.04139999999999999930055949448615137953311204910278320
mas_gorda_kg	lifestyle_mean	60	-0.259	[-0.473, -0.009]	0.04540000000000000285327317328665230888873338699340820
environment_1	imc1	80	0.224	[-0.009, 0.436]	0.04560000000000000164313007644523167982697486877441406
lean_bmc_total_kg	lev_set_cad_1	80	-0.224	[-0.426, -0.005]	0.04580000000000000043298697960381105076521635055541992
min_step_1	sent_cam_sent_1	80	-0.224	[-0.422, -0.009]	0.04619999999999999801270078592096979264169931411743164
t_score_1	alc_atr_cos_1	80	-0.223	[-0.425, -0.014]	0.04660000000000000253130849614535691216588020324707031
imc1	bmc_total_1	80	0.222	[0.011, 0.417]	0.04760000000000000341948691584548214450478553771972656
z_score_1	lean_bmc_total_kg	80	0.221	[0, 0.438]	0.04929999999999999660271754464702098630368709564208984
sedentary_mean	light_mean	60	-0.255	[-0.499, 0.021]	0.04939999999999999946709294817992486059665679931640625
moderate_mean	flex_ant_1	60	0.255	[-0.014, 0.505]	0.04939999999999999946709294817992486059665679931640625
mas_gorda_kg	veryvig_mean	60	0.252	[0.079, 0.413]	0.05220000000000000334177130412172118667513132095336914
lifestyle_mean	min_step_1	60	0.251	[-0.022, 0.492]	0.05269999999999999684696661006455542519688606262207031
min_step_1	sent_alc_1	80	0.217	[-0.002, 0.427]	0.05360000000000000180966353013900516089051961898803711
mas_gorda_kg	light_mean	60	-0.250	[-0.458, -0.022]	0.05419999999999999817923423961474327370524406433105469
mas_gorda_kg	sent_cam_sent_1	80	0.216	[-0.006, 0.427]	0.05430000000000000104360964314764714799821376800537109

Variable_1	Variable_2	n	Spearman ρ	95% CI	p-value
lean_total_kg	lev_set_cad_1	80	-0.216	[-0.421, 0.005]	0.054399999999999996909114277332264464348554611206055
vigorous_mean	sent_cam_sent_1	60	0.247	[-0.016, 0.485]	0.057299999999999996769250998340794467367231845855712 89
sedentary_mean	lev_set_cad_1	60	-0.243	[-0.478, -0.001]	0.061800000000000000766053886991358012892305850982666 02
bmd_total_1	flex_ant_1	80	0.209	[0, 0.39]	0.062600000000000002864375403532903874292969703674316 41
environment_1	bmd_total_1	80	-0.207	[-0.41, 0.023]	0.065399999999999999800159855567471822723746299743652 34
z_score_1	vigorous_mean	60	0.237	[-0.012, 0.47]	0.068599999999999994315658113919198513031005859375000 00
physical_health_1	z_score_1	80	0.204	[-0.022, 0.413]	0.068900000000000002908784324517910135909914970397949 22
t_score_1	veryvig_mean	60	0.232	[0.048, 0.406]	0.074300000000000004929390229335695039480924606323242 19
physical_health_1	lean_bmc_total_kg	80	0.196	[-0.019, 0.402]	0.081000000000000002553512956637860042974352836608886 72
bmc_total_1	veryvig_mean	60	0.224	[0.049, 0.383]	0.084800000000000000377475828372553223744034767150878 91
lifestyle_mean	moderate_mean	60	0.222	[-0.051, 0.473]	0.087700000000000000177635683940025046467781066894531 25
t_score_1	vigorous_mean	60	0.221	[-0.037, 0.459]	0.089499999999999996225241716274467762559652328491210 94
environment_1	bmc_total_1	80	-0.188	[-0.374, 0.032]	0.095299999999999995825561427409411408007144927978515 62
bmd_total_1	veryvig_mean	60	0.217	[0.019, 0.399]	0.096100000000000004862776847858185647055506706237792 97
social_relantionships_1	imc1	80	0.187	[-0.036, 0.398]	0.097199999999999994737542863276757998391985893249511 72
sedentary_mean	veryvig_mean	60	-0.216	[-0.359, -0.079]	0.098199999999999995625721282976883230730891227722167 97
imc1	lev_set_cad_1	80	-0.186	[-0.409, 0.048]	0.099199999999999996513899702677008463069796562194824 22

Variable_1	Variable_2	n	Spearman ρ	95% CI	p-value
lean_total_kg	moderate_mean	60	0.214	[-0.06, 0.465]	0.10100000000000000643929354282590793445706367492675781
area_total_1	lev_set_cad_1	80	-0.184	[-0.392, 0.026]	0.10199999999999999344968415471157641150057315826416016
t_score_1	moderate_mean	60	0.213	[-0.036, 0.446]	0.10199999999999999344968415471157641150057315826416016
moderate_mean	lev_set_cad_1	60	0.213	[-0.048, 0.45]	0.10199999999999999344968415471157641150057315826416016
area_total_1	alc_atr_cos_1	80	-0.184	[-0.395, 0.048]	0.10299999999999999433786257441170164383947849273681641
imc1	sent_cam_sent_1	80	0.183	[-0.043, 0.399]	0.10399999999999999522604099411182687617838382720947266
z_score_1	alc_atr_cos_1	80	-0.182	[-0.393, 0.047]	0.10599999999999999700239783351207734085619449615478516
mas_gorda_kg	alc_atr_cos_1	80	-0.182	[-0.381, 0.034]	0.10699999999999999789057625321220257319509983062744141
bmd_total_1	sent_cam_sent_1	80	-0.179	[-0.37, 0.033]	0.11300000000000000321964677141295396722853183746337891
mas_corp_pre	z_score_1	80	0.177	[-0.049, 0.388]	0.11600000000000000588418203051332966424524784088134766
bmc_total_1	min_step_1	80	0.177	[-0.045, 0.385]	0.11700000000000000677236045021345489658415317535400391
environment_1	alc_atr_cos_1	80	-0.176	[-0.402, 0.055]	0.11799999999999999378275106209912337362766265869140625
imc1	veryvig_mean	60	0.203	[0.019, 0.378]	0.12099999999999999644728632119949907064437866210937500
total_mass_total_kg	sent_alc_1	80	-0.173	[-0.385, 0.051]	0.12500000000000000000000000000000000000000000000000000
z_score_1	veryvig_mean	60	0.200	[-0.027, 0.392]	0.12600000000000000088817841970012523233890533447265625
veryvig_mean	sent_cam_sent_1	60	-0.199	[-0.365, -0.015]	0.12700000000000000177635683940025046467781066894531250
lean_bmc_total_kg	moderate_mean	60	0.192	[-0.068, 0.441]	0.14099999999999998645527909957309020683169364929199219

Variable_1	Variable_2	n	Spearman ρ	95% CI	p-value
physical_health_1	sent_cam_sent_1	80	-0.165	[-0.368, 0.063]	0.14399999999999998911981435867346590384840965270996094
social_relantionships_1	total_mass_total_kg	80	0.165	[-0.051, 0.379]	0.14399999999999998911981435867346590384840965270996094
light_mean	sent_alc_1	60	0.191	[-0.082, 0.445]	0.14499999999999999000799277837359113618731498718261719
z_score_1	lean_total_kg	80	0.164	[-0.054, 0.387]	0.14699999999999999178434961777384160086512565612792969
lifestyle_mean	flex_ant_1	60	0.190	[-0.068, 0.431]	0.14699999999999999178434961777384160086512565612792969
lifestyle_mean	sent_cam_sent_1	60	-0.188	[-0.431, 0.073]	0.15099999999999999533706329657434253022074699401855469
lean_bmc_total_kg	lifestyle_mean	60	-0.187	[-0.432, 0.068]	0.15199999999999999622524171627446776255965232849121094
bmc_total_1	moderate_mean	60	0.186	[-0.075, 0.428]	0.15499999999999999888977697537484345957636833190917969
psychological_1	veryvig_mean	60	0.185	[-0.024, 0.38]	0.15599999999999999977795539507496869191527366638183594
psychological_1	area_total_1	80	0.160	[-0.048, 0.351]	0.15700000000000000066613381477509392425417900085449219
physical_health_1	mas_corp_pre	80	0.158	[-0.068, 0.387]	0.16300000000000000599520433297584531828761100769042969
psychological_1	mas_corp_pre	80	0.156	[-0.058, 0.367]	0.16700000000000000954791801177634624764323234558105469
sent_alc_1	sent_cam_sent_1	80	-0.156	[-0.358, 0.05]	0.16700000000000000954791801177634624764323234558105469
bmc_total_1	sent_cam_sent_1	80	-0.156	[-0.36, 0.055]	0.16800000000000001043609643147647147998213768005371094
psychological_1	total_mass_total_kg	80	0.155	[-0.06, 0.358]	0.16900000000000001132427485117659671232104301452636719
lean_total_kg	lifestyle_mean	60	-0.180	[-0.417, 0.079]	0.16900000000000001132427485117659671232104301452636719
psychological_1	social_relantionships_1	80	-0.155	[-0.376, 0.075]	0.17000000000000001221245327087672194465994834899902344

Variable_1	Variable_2	n	Spearman ρ	95% CI	p-value
mas_corp_pre	min_step_1	80	-0.155	[-0.354, 0.053]	0.17000000000000001221245327087672194465994834899902344
environment_1	area_total_1	80	-0.154	[-0.364, 0.071]	0.17199999999999998623323449464805889874696731567382812
lean_bmc_total_kg	sent_alc_1	80	-0.152	[-0.354, 0.068]	0.17799999999999999156230501284881029278039932250976562
alc_atr_cos_1	sent_cam_sent_1	80	-0.152	[-0.373, 0.083]	0.17799999999999999156230501284881029278039932250976562
lifestyle_mean	sent_alc_1	60	0.176	[-0.108, 0.426]	0.17899999999999999245048343254893552511930465698242188
area_total_1	vigorous_mean	60	0.172	[-0.087, 0.421]	0.18800000000000000044408920985006261616945266723632812
light_mean	sent_cam_sent_1	60	-0.171	[-0.426, 0.088]	0.19200000000000000399680288865056354552507400512695312
physical_health_1	lev_set_cad_1	80	0.147	[-0.099, 0.369]	0.19400000000000000577315972805081401020288467407226562
vigorous_mean	alc_atr_cos_1	60	-0.170	[-0.409, 0.102]	0.19400000000000000577315972805081401020288467407226562
mas_corp_pre	flex_ant_1	80	-0.146	[-0.355, 0.059]	0.19700000000000000843769498715118970721960067749023438
mas_corp_pre	sent_alc_1	80	-0.145	[-0.355, 0.072]	0.19900000000000001021405182655144017189741134643554688
physical_health_1	total_mass_total_kg	80	0.145	[-0.083, 0.368]	0.20000000000000001110223024625156540423631668090820312
physical_health_1	flex_ant_1	80	0.145	[-0.079, 0.381]	0.20000000000000001110223024625156540423631668090820312
psychological_1	bmc_total_1	80	0.142	[-0.071, 0.349]	0.20899999999999999134026040792377898469567298889160156
mas_corp_pre	veryvig_mean	60	0.163	[-0.08, 0.378]	0.21199999999999999400479566702415468171238899230957031
social_relantionships_1	flex_ant_1	80	0.140	[-0.065, 0.324]	0.21699999999999999844568776552478084340691566467285156
bmc_total_1	vigorous_mean	60	0.161	[-0.105, 0.419]	0.22000000000000000111022302462515654042363166809082031

Variable_1	Variable_2	n	Spearman ρ	95% CI	p-value
mas_gorda_kg	lean_bmc_total_kg	80	0.138	[-0.082, 0.35]	0.22300000000000000377475828372553223744034767150878906
physical_health_1	lean_total_kg	80	0.137	[-0.075, 0.353]	0.22700000000000000732747196252603316679596900939941406
bmc_total_1	alc_atr_cos_1	80	-0.136	[-0.361, 0.093]	0.22700000000000000732747196252603316679596900939941406
psychological_1	lean_bmc_total_kg	80	0.136	[-0.07, 0.337]	0.22900000000000000910382880192628363147377967834472656
environment_1	flex_ant_1	80	0.135	[-0.107, 0.358]	0.23300000000000001265654248072678456082940101623535156
imc1	t_score_1	80	0.133	[-0.083, 0.335]	0.23999999999999999111821580299874767661094665527343750
z_score_1	mas_gorda_kg	80	0.133	[-0.118, 0.356]	0.23999999999999999111821580299874767661094665527343750
psychological_1	imc1	80	0.132	[-0.089, 0.352]	0.24199999999999999289457264239899814128875732421875000
social_relantionships_1	moderate_mean	60	-0.152	[-0.372, 0.081]	0.24599999999999999644728632119949907064437866210937500
z_score_1	total_mass_total_kg	80	0.131	[-0.11, 0.351]	0.24599999999999999644728632119949907064437866210937500
lean_total_kg	flex_ant_1	80	-0.131	[-0.339, 0.076]	0.24599999999999999644728632119949907064437866210937500
lifestyle_mean	veryvig_mean	60	-0.152	[-0.365, 0.071]	0.24699999999999999733546474089962430298328399658203125
total_mass_total_kg	flex_ant_1	80	-0.130	[-0.34, 0.084]	0.25000000000000000000000000000000000000000000000000000
physical_health_1	mas_gorda_kg	80	0.126	[-0.09, 0.319]	0.26700000000000001509903313490212894976139068603515625
social_relantionships_1	alc_atr_cos_1	80	-0.126	[-0.339, 0.091]	0.26700000000000001509903313490212894976139068603515625
social_relantionships_1	light_mean	60	-0.145	[-0.391, 0.115]	0.27000000000000001776356839400250464677810668945312500

Variable_1	Variable_2	n	Spearman ρ	95% CI	p-value
mas_gorda_kg	flex_ant_1	80	0.125	[-0.085, 0.321]	0.27000000000000001776356839400250464677810668945312500
veryvig_mean	flex_ant_1	60	0.144	[-0.087, 0.378]	0.27300000000000002042810365310288034379482269287109375
physical_health_1	sedentary_mean	60	0.144	[-0.115, 0.391]	0.27400000000000002131628207280300557613372802734375000
mas_gorda_kg	min_step_1	80	-0.123	[-0.331, 0.09]	0.27800000000000002486899575160350650548934936523437500
imc1	flex_ant_1	80	-0.122	[-0.328, 0.094]	0.28000000000000002664535259100375697016716003417968750
physical_health_1	alc_atr_cos_1	80	-0.121	[-0.355, 0.099]	0.28399999999999997468691503854643087834119796752929688
total_mass_total_kg	min_step_1	80	-0.119	[-0.321, 0.087]	0.29199999999999998179234239614743273705244064331054688
psychological_1	lean_total_kg	80	0.119	[-0.091, 0.332]	0.29399999999999998356869923554768320173025131225585938
bmc_total_1	flex_ant_1	80	0.119	[-0.085, 0.305]	0.29399999999999998356869923554768320173025131225585938
z_score_1	flex_ant_1	80	0.118	[-0.102, 0.32]	0.29499999999999998445687765524780843406915664672851562
lean_total_kg	sent_alc_1	80	-0.118	[-0.329, 0.097]	0.29599999999999998534505607494793366640806198120117188
sedentary_mean	min_step_1	60	-0.136	[-0.369, 0.113]	0.30099999999999998978594817344855982810258865356445312
environment_1	sent_cam_sent_1	80	0.116	[-0.116, 0.337]	0.30599999999999999422684027194918598979711532592773438
lifestyle_mean	alc_atr_cos_1	60	0.133	[-0.118, 0.36]	0.30999999999999999777955395074968691915273666381835938
moderate_mean	alc_atr_cos_1	60	-0.133	[-0.375, 0.122]	0.30999999999999999777955395074968691915273666381835938
z_score_1	moderate_mean	60	0.133	[-0.121, 0.366]	0.31099999999999999866773237044981215149164199829101562
social_relantionships_1	sedentary_mean	60	-0.132	[-0.401, 0.151]	0.31400000000000000133226762955018784850835800170898438

Variable_1	Variable_2	n	Spearman ρ	95% CI	p-value
imc1	bmd_total_1	80	0.114	[-0.103, 0.337]	0.3160000000000000310862446895043831318616867065429688
social_relantionships_1	lev_set_cad_1	80	0.113	[-0.119, 0.338]	0.31800000000000000488498130835068877786397933959960938
area_total_1	veryvig_mean	60	0.131	[-0.008, 0.283]	0.31800000000000000488498130835068877786397933959960938
area_total_1	min_step_1	80	0.113	[-0.116, 0.335]	0.31900000000000000577315972805081401020288467407226562
area_total_1	sent_alc_1	80	-0.112	[-0.322, 0.11]	0.32100000000000000754951656745106447488069534301757812
mas_gorda_kg	lean_total_kg	80	0.112	[-0.1, 0.327]	0.32100000000000000754951656745106447488069534301757812
area_total_1	mas_gorda_kg	80	0.112	[-0.11, 0.33]	0.32300000000000000932587340685131493955850601196289062
lean_bmc_total_kg	flex_ant_1	80	-0.110	[-0.312, 0.095]	0.33400000000000001909583602355269249528646469116210938
alc_atr_cos_1	lev_set_cad_1	80	0.109	[-0.121, 0.325]	0.33500000000000001998401444325281772762537002563476562
mas_gorda_kg	vigorous_mean	60	0.124	[-0.14, 0.376]	0.34399999999999997246646898929611779749393463134765625
mas_gorda_kg	sedentary_mean	60	-0.124	[-0.369, 0.122]	0.34599999999999997424282582869636826217174530029296875
lifestyle_mean	vigorous_mean	60	-0.124	[-0.393, 0.168]	0.34599999999999997424282582869636826217174530029296875
environment_1	moderate_mean	60	-0.123	[-0.371, 0.145]	0.34999999999999997779553950749686919152736663818359375
social_relantionships_1	sent_cam_sent_1	80	0.105	[-0.11, 0.31]	0.35599999999999998312461002569762058556079864501953125
social_relantionships_1	t_score_1	80	0.104	[-0.134, 0.316]	0.35899999999999998578914528479799628257751464843750000
social_relantionships_1	sent_alc_1	80	-0.104	[-0.316, 0.109]	0.36099999999999998756550212419824674725532531738281250

Variable_1	Variable_2	n	Spearman ρ	95% CI	p-value
environment_1	vigorous_mean	60	-0.116	[-0.351, 0.132]	0.37600000000000000088817841970012523233890533447265625
t_score_1	min_step_1	80	0.100	[-0.137, 0.312]	0.378000000000000002664535259100375697016716003417968750
imc1	moderate_mean	60	-0.115	[-0.366, 0.151]	0.38000000000000000444089209850062616169452667236328125
area_total_1	sent_cam_sent_1	80	-0.098	[-0.312, 0.116]	0.387000000000000010658141036401502788066864013671875000
bmd_total_1	lifestyle_mean	60	-0.112	[-0.366, 0.159]	0.393000000000000015987211554602254182100296020507812500
environment_1	mas_corp_pre	80	0.095	[-0.143, 0.314]	0.403000000000000024868995751603506505489349365234375000
moderate_mean	min_step_1	60	0.110	[-0.138, 0.359]	0.403000000000000024868995751603506505489349365234375000
psychological_1	min_step_1	80	-0.094	[-0.32, 0.139]	0.405000000000000026645352591003756970167160034179687500
bmc_total_1	mas_gorda_kg	80	0.094	[-0.13, 0.318]	0.405000000000000026645352591003756970167160034179687500
lean_bmc_total_kg	veryvig_mean	60	0.108	[-0.117, 0.309]	0.409999999999999975575093458246556110680103302001953125
lean_bmc_total_kg	vigorous_mean	60	0.107	[-0.147, 0.356]	0.413999999999999979127807137047057040035724639892578125
bmd_total_1	vigorous_mean	60	0.107	[-0.167, 0.386]	0.415999999999999980904163976447307504713535308837890625
t_score_1	sent_cam_sent_1	80	-0.091	[-0.304, 0.117]	0.421999999999999986233234494648058898746967315673828125
social_relantionshi ps_1	bmc_total_1	80	-0.091	[-0.304, 0.137]	0.423999999999999988009591334048309363424777984619140625
area_total_1	light_mean	60	0.105	[-0.181, 0.391]	0.424999999999999988897769753748434595763683319091796875
social_relantionshi ps_1	area_total_1	80	-0.089	[-0.307, 0.144]	0.429999999999999993338661852249060757458209991455078125
bmc_total_1	lifestyle_mean	60	-0.104	[-0.357, 0.158]	0.430999999999999994226840271949185989797115325927734375

Variable_1	Variable_2	n	Spearman ρ	95% CI	p-value
psychological_1	t_score_1	80	0.088	[-0.133, 0.302]	0.4390000000000000013322676295501878485083580017089843 8
veryvig_mean	lev_set_cad_1	60	0.101	[-0.2, 0.378]	0.44400000000000000577315972805081401020288467407226562
bmc_total_1	lev_set_cad_1	80	-0.086	[-0.298, 0.129]	0.44700000000000000843769498715118970721960067749023438
social_relantionships_1	mas_corp_pre	80	0.084	[-0.152, 0.31]	0.45800000000000001820765760385256726294755935668945312
lean_total_kg	vigorous_mean	60	0.097	[-0.153, 0.342]	0.45900000000000001909583602355269249528646469116210938
total_mass_total_kg	sent_cam_sent_1	80	0.084	[-0.142, 0.292]	0.46100000000000002087219286295294295996427536010742188
psychological_1	lifestyle_mean	60	0.097	[-0.189, 0.379]	0.46200000000000002176037128265306819230318069458007812
z_score_1	min_step_1	80	0.083	[-0.149, 0.295]	0.46400000000000002353672812205331865698099136352539062
psychological_1	sedentary_mean	60	0.095	[-0.184, 0.378]	0.46800000000000002708944180085381958633661270141601562
psychological_1	sent_alc_1	80	0.081	[-0.127, 0.29]	0.47499999999999997779553950749686919152736663818359375
alc_atr_cos_1	sent_alc_1	80	0.081	[-0.119, 0.288]	0.47499999999999997779553950749686919152736663818359375
psychological_1	moderate_mean	60	-0.092	[-0.376, 0.187]	0.48499999999999998667732370449812151491641998291015625
sedentary_mean	alc_atr_cos_1	60	0.092	[-0.15, 0.355]	0.48499999999999998667732370449812151491641998291015625
social_relantionships_1	min_step_1	80	-0.077	[-0.293, 0.154]	0.49699999999999999733546474089962430298328399658203125
mas_corp_pre	sent_cam_sent_1	80	0.076	[-0.146, 0.286]	0.50000000000000000000000000000000000000000000000000000
mas_corp_pre	vigorous_mean	60	0.087	[-0.175, 0.339]	0.50700000000000000621724893790087662637233734130859375

Variable_1	Variable_2	n	Spearman ρ	95% CI	p-value
psychological_1	mas_gorda_kg	80	0.073	[-0.141, 0.293]	0.520000000000000017763568394002504646778106689453125 00
environment_1	total_mass_total_kg	80	0.073	[-0.164, 0.296]	0.52300000000000002042810365310288034379482269287109375
social_relantionships_1	bmd_total_1	80	-0.072	[-0.28, 0.141]	0.52400000000000002131628207280300557613372802734375000
environment_1	light_mean	60	-0.084	[-0.327, 0.172]	0.52400000000000002131628207280300557613372802734375000
lean_total_kg	light_mean	60	-0.083	[-0.351, 0.197]	0.53000000000000002664535259100375697016716003417968750
environment_1	sedentary_mean	60	0.082	[-0.177, 0.332]	0.53400000000000003019806626980425789952278137207031250
lean_bmc_total_kg	light_mean	60	-0.081	[-0.347, 0.213]	0.53700000000000003286260152890463359653949737548828125
lean_total_kg	sent_cam_sent_1	80	-0.069	[-0.279, 0.14]	0.54100000000000003641531520770513452589511871337890625
t_score_1	light_mean	60	0.080	[-0.186, 0.338]	0.54200000000000003730349362740525975823402404785156250
area_total_1	lifestyle_mean	60	-0.080	[-0.328, 0.184]	0.54400000000000003907985046680551022291183471679687500
bmd_total_1	mas_gorda_kg	80	0.069	[-0.156, 0.286]	0.54600000000000004085620730620576068758964538574218750
light_mean	veryvig_mean	60	-0.078	[-0.314, 0.149]	0.55500000000000004884981308350688777863979339599609375
psychological_1	vigorous_mean	60	-0.075	[-0.322, 0.175]	0.56699999999999994848565165739273652434349060058593750
vigorous_mean	sent_alc_1	60	-0.075	[-0.325, 0.178]	0.56999999999999995115018691649311222136020660400390625
psychological_1	lev_set_cad_1	80	-0.063	[-0.293, 0.167]	0.58099999999999996092014953319448977708816528320312500
physical_health_1	imc1	80	0.062	[-0.165, 0.289]	0.58399999999999996358468479229486547410488128662109375
moderate_mean	sent_cam_sent_1	60	-0.070	[-0.318, 0.174]	0.59299999999999997157829056959599256515502929687500000

Variable_1	Variable_2	n	Spearman ρ	95% CI	p-value
social_relantionships_1	vigorous_mean	60	-0.070	[-0.327, 0.186]	0.59699999999999997513100424839649349451065063476562500
area_total_1	sedentary_mean	60	-0.070	[-0.325, 0.207]	0.59699999999999997513100424839649349451065063476562500
mas_corp_pre	moderate_mean	60	0.068	[-0.177, 0.314]	0.60499999999999998223643160599749535322189331054687500
moderate_mean	sent_alc_1	60	0.067	[-0.197, 0.323]	0.60799999999999998490096686509787105023860931396484375
t_score_1	lev_set_cad_1	80	0.057	[-0.164, 0.263]	0.61599999999999999200639422269887290894985198974609375
t_score_1	sent_alc_1	80	-0.057	[-0.312, 0.193]	0.61599999999999999200639422269887290894985198974609375
bmd_total_1	lev_set_cad_1	80	0.055	[-0.169, 0.256]	0.62800000000000000266453525910037569701671600341796875
physical_health_1	social_relantionships_1	80	-0.054	[-0.285, 0.18]	0.63500000000000000888178419700125232338905334472656250
social_relantionships_1	lifestyle_mean	60	-0.063	[-0.333, 0.21]	0.63500000000000000888178419700125232338905334472656250
lean_bmc_total_kg	sent_cam_sent_1	80	-0.052	[-0.26, 0.158]	0.64800000000000002042810365310288034379482269287109375
physical_health_1	light_mean	60	0.060	[-0.218, 0.328]	0.64900000000000002131628207280300557613372802734375000
lean_total_kg	veryvig_mean	60	0.060	[-0.179, 0.284]	0.65100000000000002309263891220325604081153869628906250
physical_health_1	moderate_mean	60	0.058	[-0.215, 0.326]	0.66200000000000003286260152890463359653949737548828125
z_score_1	light_mean	60	0.057	[-0.209, 0.322]	0.66300000000000003375077994860475882887840270996093750
sedentary_mean	sent_cam_sent_1	60	-0.057	[-0.308, 0.2]	0.66400000000000003463895836830488406121730804443359375
z_score_1	sent_alc_1	80	-0.049	[-0.295, 0.197]	0.66800000000000003819167204710538499057292938232421875
lean_bmc_total_kg	sedentary_mean	60	0.056	[-0.214, 0.32]	0.67100000000000004085620730620576068758964538574218750

Variable_1	Variable_2	n	Spearman ρ	95% CI	p-value
bmc_total_1	sent_alc_1	80	-0.048	[-0.262, 0.173]	0.67300000000000004263256414560601115226745605468750000
light_mean	alc_atr_cos_1	60	0.056	[-0.201, 0.313]	0.67300000000000004263256414560601115226745605468750000
vigorous_mean	flex_ant_1	60	0.055	[-0.2, 0.304]	0.67500000000000004440892098500626161694526672363281250
z_score_1	lev_set_cad_1	80	-0.045	[-0.266, 0.16]	0.68999999999999994670929481799248605966567993164062500
psychological_1	light_mean	60	0.051	[-0.231, 0.324]	0.69799999999999995381472217559348791837692260742187500
total_mass_total_kg	veryvig_mean	60	-0.051	[-0.264, 0.182]	0.69899999999999995470290059529361315071582794189453125
physical_health_1	sent_alc_1	80	-0.043	[-0.247, 0.174]	0.70399999999999995914379269379423931241035461425781250
bmd_total_1	sedentary_mean	60	0.049	[-0.214, 0.314]	0.70799999999999996269650637259474024176597595214843750
environment_1	lean_bmc_total_kg	80	-0.042	[-0.268, 0.191]	0.70899999999999996358468479229486547410488128662109375
vigorous_mean	lev_set_cad_1	60	-0.049	[-0.297, 0.229]	0.70999999999999996447286321199499070644378662109375000
imc1	vigorous_mean	60	0.048	[-0.224, 0.317]	0.71399999999999996802557689079549163579940795898437500
imc1	sedentary_mean	60	-0.048	[-0.319, 0.216]	0.71599999999999996980193373019574210047721862792968750
social_relantionships_1	veryvig_mean	60	0.047	[-0.285, 0.318]	0.72099999999999997424282582869636826217174530029296875
bmd_total_1	alc_atr_cos_1	80	-0.040	[-0.274, 0.202]	0.72599999999999997868371792719699442386627197265625000
t_score_1	lifestyle_mean	60	-0.046	[-0.3, 0.214]	0.72599999999999997868371792719699442386627197265625000
environment_1	lean_total_kg	80	-0.039	[-0.267, 0.194]	0.73299999999999998490096686509787105023860931396484375
bmc_total_1	light_mean	60	0.044	[-0.241, 0.344]	0.73699999999999998845368054389837197959423065185546875

Variable_1	Variable_2	n	Spearman ρ	95% CI	p-value
social_relantionships_1	lean_total_kg	80	-0.037	[-0.255, 0.19]	0.74199999999999999289457264239899814128875732421875000
mas_gorda_kg	lev_set_cad_1	80	-0.037	[-0.261, 0.193]	0.74799999999999999822364316059974953532218933105468750
z_score_1	sedentary_mean	60	0.042	[-0.199, 0.285]	0.75100000000000000088817841970012523233890533447265625
total_mass_total_kg	moderate_mean	60	0.041	[-0.205, 0.295]	0.75400000000000000355271367880050092935562133789062500
psychological_1	flex_ant_1	80	-0.033	[-0.258, 0.199]	0.76800000000000001598721155460225418210029602050781250
z_score_1	lifestyle_mean	60	-0.038	[-0.273, 0.212]	0.77400000000000002131628207280300557613372802734375000
veryvig_mean	sent_alc_1	60	0.038	[-0.087, 0.177]	0.77400000000000002131628207280300557613372802734375000
bmd_total_1	sent_alc_1	80	0.032	[-0.186, 0.26]	0.77900000000000002575717417130363173782825469970703125
physical_health_1	lifestyle_mean	60	0.036	[-0.24, 0.306]	0.78300000000000002930988785010413266718387603759765625
imc1	sent_alc_1	80	-0.029	[-0.244, 0.194]	0.80200000000000004618527782440651208162307739257812500
environment_1	lev_set_cad_1	80	0.028	[-0.218, 0.272]	0.80800000000000005151434834260726347565650939941406250
light_mean	vigorous_mean	60	0.031	[-0.244, 0.319]	0.81200000000000005506706202140776440501213073730468750
bmd_total_1	moderate_mean	60	0.030	[-0.233, 0.307]	0.81799999999999994937383007709286175668239593505859375
psychological_1	bmd_total_1	80	0.026	[-0.184, 0.244]	0.81999999999999995115018691649311222136020660400390625
social_relantionships_1	z_score_1	80	0.025	[-0.203, 0.235]	0.82499999999999995559107901499373838305473327636718750
lean_total_kg	min_step_1	80	0.025	[-0.196, 0.244]	0.82599999999999995647925743469386361539363861083984375

Variable_1	Variable_2	n	Spearman ρ	95% CI	p-value
environment_1	lifestyle_mean	60	0.029	[-0.257, 0.3]	0.82799999999999995825561427409411408007144927978515625
total_mass_total_kg	vigorous_mean	60	0.028	[-0.229, 0.267]	0.83099999999999996092014953319448977708816528320312500
bmc_total_1	sedentary_mean	60	-0.027	[-0.283, 0.235]	0.83599999999999996536104163169511593878269195556640625
area_total_1	flex_ant_1	80	0.023	[-0.177, 0.228]	0.83699999999999996624922005139524117112159729003906250
veryvig_mean	alc_atr_cos_1	60	0.026	[-0.173, 0.28]	0.84199999999999997069011214989586733281612396240234375
vigorous_mean	min_step_1	60	0.026	[-0.241, 0.313]	0.84399999999999997246646898929611779749393463134765625
total_mass_total_kg	sedentary_mean	60	0.026	[-0.249, 0.294]	0.84499999999999997335464740899624302983283996582031250
physical_health_1	vigorous_mean	60	0.025	[-0.23, 0.277]	0.84699999999999997513100424839649349451065063476562500
lean_bmc_total_kg	min_step_1	80	0.022	[-0.205, 0.238]	0.84899999999999997690736108779674395918846130371093750
lean_total_kg	sedentary_mean	60	0.024	[-0.238, 0.294]	0.85599999999999998312461002569762058556079864501953125
imc1	z_score_1	80	-0.020	[-0.246, 0.191]	0.85799999999999998490096686509787105023860931396484375
social_relantionships_1	lean_bmc_total_kg	80	-0.019	[-0.242, 0.211]	0.86399999999999999023003738329862244427204132080078125
environment_1	veryvig_mean	60	-0.022	[-0.213, 0.157]	0.86999999999999999555910790149937383830547332763671875
environment_1	z_score_1	80	-0.017	[-0.229, 0.206]	0.87900000000000000355271367880050092935562133789062500
mas_gorda_kg	moderate_mean	60	0.020	[-0.231, 0.304]	0.87900000000000000355271367880050092935562133789062500
psychological_1	sent_cam_sent_1	80	0.016	[-0.214, 0.248]	0.88900000000000001243449787580175325274467468261718750

Variable_1	Variable_2	n	Spearman ρ	95% CI	p-value
veryvig_mean	min_step_1	60	-0.018	[-0.243, 0.166]	0.89200000000000001509903313490212894976139068603515625
environment_1	sent_alc_1	80	0.015	[-0.21, 0.229]	0.89700000000000001953992523340275511145591735839843750
alc_atr_cos_1	flex_ant_1	80	0.015	[-0.224, 0.244]	0.89700000000000001953992523340275511145591735839843750
bmd_total_1	light_mean	60	-0.016	[-0.292, 0.27]	0.90300000000000002486899575160350650548934936523437500
t_score_1	sedentary_mean	60	-0.014	[-0.257, 0.241]	0.91400000000000003463895836830488406121730804443359375
mas_gorda_kg	sent_alc_1	80	0.011	[-0.225, 0.24]	0.92000000000000003996802888650563545525074005126953125
environment_1	min_step_1	80	-0.011	[-0.252, 0.22]	0.92100000000000004085620730620576068758964538574218750
mas_corp_pre	sedentary_mean	60	0.013	[-0.262, 0.282]	0.92300000000000004263256414560601115226745605468750000
physical_health_1	min_step_1	80	0.011	[-0.216, 0.247]	0.92400000000000004352074256530613638460636138916015625
psychological_1	alc_atr_cos_1	80	0.011	[-0.216, 0.221]	0.92400000000000004352074256530613638460636138916015625
environment_1	t_score_1	80	0.010	[-0.215, 0.234]	0.93100000000000004973799150320701301097869873046875000
z_score_1	sent_cam_sent_1	80	0.007	[-0.208, 0.223]	0.95299999999999995825561427409411408007144927978515625
psychological_1	z_score_1	80	-0.003	[-0.233, 0.228]	0.97599999999999997868371792719699442386627197265625000
moderate_mean	veryvig_mean	60	-0.004	[-0.3, 0.236]	0.97599999999999997868371792719699442386627197265625000
sedentary_mean	lifestyle_mean	60	-0.003	[-0.269, 0.273]	0.98199999999999998401278844539774581789970397949218750
alc_atr_cos_1	min_step_1	80	0.002	[-0.228, 0.235]	0.98699999999999998845368054389837197959423065185546875
